# Perceived Discrimination and Aggression Among Chinese Migrant Adolescents: A Moderated Mediation Model

**DOI:** 10.3389/fpsyg.2021.651270

**Published:** 2021-03-03

**Authors:** Ruoshan Xiong, Yiwei Xia, Spencer D. Li

**Affiliations:** ^1^Department of Social Work, College of Humanities and Social Sciences, Huazhong Agricultural University, Wuhan, China; ^2^School of Law, Southwestern University of Finance and Economics, Chengdu, China; ^3^Department of Sociology, Faculty of Social Sciences, University of Macau, Macao, China

**Keywords:** perceived discrimination, aggression, negative emotions, socioemotional support, migrant adolescents

## Abstract

Previous research has showed that Chinese rural-to-urban migrant adolescents are at high risk for discrimination, negative emotions, and aggression. However, little is known about how discrimination, negative emotions, and aggression are interrelated and whether social support addressing the emotional needs of the adolescents would moderate the relationship of discrimination to aggression. This study attempts to fill these gaps. Based on prior research, it is proposed that perceived discrimination relates to reactive aggression by increasing negative emotions that foster aggressive responses to stressful events. Considering the central role that negative emotions may play, it is also hypothesized that socioemotional support provided by family, friends, and community mitigates the impact of perceived discrimination on reactive aggression by reducing negative emotions. The results obtained from the analysis of two-wave survey data collected from a probability sample of 470 migrant students aged 11–17 (46.17% female; mean age = 13.49) in China supported these hypotheses. The findings indicate that perceived discrimination fosters negative emotions, which in turn increase reactive aggression. Additionally, socioemotional support reduces the adverse impact of perceived discrimination on reactive aggression by weakening the link between perceived discrimination and negative emotions. Practical and policy implications of these findings are discussed.

## Introduction

Aggression is a frequently observed behavior among children and adolescents (Connor, 2002; Hartshorn et al., 2012). Children of migrants, including domestic and international migrants, are especially at risk for aggression (WHO Regional Office for Europe, 2010; Li and Xia, 2018). Some studies have shown that over 50% of migrant adolescents experienced interpersonal conflicts related to peer aggression (Qin et al., 2008). Research has linked the high prevalence rate to discrimination experienced by these children in the receiving places (Lan et al., 2020; Song et al., 2020). They argued that discrimination impedes the development of these children by increasing their involvement in antisocial behavior.

The existing research has generally treated discrimination as a precursor to aggression (Wright and Wachs, 2019; Mulvey et al., 2020; Xie et al., 2020). However, aggression is a complex and multifaceted construct that may encompass different subtypes. Researchers have distinguished between proactive and reactive aggression (Dodge, 1991; Raine et al., 2006). Proactive aggression is a deliberate, coercive behavior used as a means of achieving a desired goal, while reactive aggression occurs as a retaliatory or defensive response to frustration or provocation and is often associated with elevated levels of emotional problems. Of these two types, reactive aggression may be more likely to occur in response to an aversive event such as discrimination and may affect migrant children more strongly (Connor, 2002; Bushman, 2017).

However, despite the emerging evidence supporting the role of discrimination in the instigation of reactive aggression, few studies have examined the relationship between discrimination and aggression. Additionally, to our knowledge, no study has explicitly tested how discrimination, negative emotions, and aggression are interrelated and whether socioemotional support addressing the emotional needs of the adolescents would moderate the effect of discrimination on aggression. This study aims to fill these gaps by proposing and empirically testing a moderated mediation model that integrates the mediating roles of negative emotions and the moderating role of socioemotional support in the relationship between perceived discrimination and adolescent aggression.

The data used to test the model were collected from a two-wave longitudinal survey of a probability sample of Chinese migrant adolescents. The experiences of migrant children in China are especially pertinent to the research questions. In the last several decades, China has undergone rapid industrialization and urbanization. As demands for factory workers and service labors grew, hundreds of millions of rural residents migrated to cities to seek better employment opportunities, and living conditions, with many of them bringing children with them. This pattern of migration has displaced nearly 36 million rural-born children and moved them to the big cities (Duan et al., 2013). It is well-documented that these migrant children have a higher tendency to engage in aggression and violence than native-born children (Zhong et al., 2017). What is less clear is what causes these children to be more heavily involved in aggressive behavior. Discrimination and its link to reactive aggression may hold one of the answers, as discrimination may lead to anger and frustration that invoke aggressive responses. In mainland China, rural migrants are often subject to discrimination due to their lower social status and the institutional barriers that deprive them of the rights afforded to urban residents (Kuang and Liu, 2012). A large amount of empirical evidence indicated that migrant adolescents in China were confronted with individual and institutional discrimination (Wang and Mesman, 2015; Lan and Moscardino, 2020). Data collected from this population present a unique opportunity to test whether perceived discrimination is related to aggression through this process.

Perceived discrimination refers to individuals' perception of negative attitude, judgment, or unfair treatment due to their specific characteristics such as gender, race, ethnicity, and social status (Banks et al., 2006). In this study, we focus specifically on discrimination related to social status. Discrimination has been identified as a predictor of aggression and violence among migrant children in many studies (Smokowski and Bacallao, 2006; Rivera et al., 2010). The general strain theory (GST) proposed by Agnew (1992) considers delinquent behavior as a strategy for coping with stressful experiences. According to the GST, strains are “relationships in which others are not treating the individual as he or she would like to be treated” (Agnew, 1992, p. 48). Such relationships create negative emotions, which in turn lead to delinquent coping that may include aggressive behavior. The GST (Agnew, 1992, 2013) contends that discrimination is one of the strains that promote delinquent coping because it is often seen as unjust and denigratory by the person who receives the unfair treatment. The theoretical underpinnings of the GST are supported by empirical research showing a positive relationship between discrimination and adolescent aggression. This relationship has emerged across different adolescent demographic groups including Latinx adolescents (Smokowski and Bacallao, 2006; Wright and Wachs, 2019), African American youth (Mulvey et al., 2020; Xie et al., 2020), and Chinese migrant adolescents (Beiser et al., 2010; Lan et al., 2020; Song et al., 2020).

Discrimination may have different effects on proactive and reactive aggression. Dodge (1991) maintained that reactive and proactive aggression arise from distinct social experiences and develop independently. The concept of reactive aggression derives from Berkowitz (1962) frustration-aggression model, which contends that reactive aggression occurs as a retaliatory or defensive response to perceived provocation or offenses and usually comes along with intense emotions of anger and frustration. This model posits that stressful life events such as exposure to discrimination that are perceived as intentional or threatening elicit feelings of fear or the need to defend oneself, thereby leading to an escalation in reactive aggression (Berkowitz, 1978).

On the contrary, proactive aggression, which is rooted in the social learning theory (Bandura, 1973), is referred to as aggressive behavior directed toward achieving a desired goal. Unlike reactive aggression that happens as a defensive reaction to situational stimulus, proactive aggression develops in supportive circumstances that value the use of aggression as an effective strategy to resolve conflicts or obtain desired outcomes (Dodge and Coie, 1987; Dodge, 1991).

As a reaction to the anger–frustration stimulus, reactive aggression is more likely to be triggered by situational factors in comparison with proactive aggression. Since discrimination is mainly a situational factor, it should affect reactive aggression more strongly. Consistent with this view, empirical literature has shown that adolescents subjected to peer rejection and provocations tend to perceive peers' intentions as hostile, leading to an increased likelihood of reactive rather than proactive aggression (Camodeca et al., 2002; Card and Little, 2006; Fite et al., 2012; Brown et al., 2016). In line with these studies, Chan et al. (2018) found that that verbal victimization by peers significantly contributes more to adolescent reactive aggression than proactive aggression in a sample of more than 1,000 Hong Kong youth. Considering this evidence, we expect that perceived discrimination is more strongly related to adolescent reactive aggression than proactive aggression.

According to the GST (Agnew, 1992, 2013), the relationship between stressful circumstances and delinquent coping is not straightforward. Rather, exposure to stressful experiences like discrimination generate negative emotions, which in turn provide major impetus for delinquent coping that may include aggressive behavior. The GST contends that negative emotions can render people irritable, impatient, resentful, and explosive. People who suffer from these emotions have a stronger desire for revenge, which may lead to the elevated risk of delinquent coping including aggression (Agnew, 1992). Indeed, negative emotions operate as intervening variables between discrimination and aggression, as evidenced in prior research demonstrating that discrimination is linked to aggression through negative emotions (Simons et al., 2006; Hartshorn et al., 2012; Herts et al., 2012).

Negative emotions have been differentially associated with reactive and proactive aggression (Vitaro et al., 2002). The frustration-anger theory of aggression contends that reactive aggression is emotionally based behavior stemming from an emotional and impulsive reaction to perceived provocation (Berkowitz, 1962, 1993). According to the theory, negative emotions especially anger and frustration lay the foundation for reactive aggression. In consistency with this argument, reactive aggression has been found to occur in the context of multiple mental health problems, especially anger and hostile emotions (Frick and Morris, 2004; Marsee and Frick, 2007; Moore et al., 2019). Children who have experienced intense and dysregulated anger tend to act out by involving in reactive aggression when provoked by situational stimuli (Damon et al., 2006). Prior research has documented the important role that anger plays as a catalyst for reactive aggression (Sullivan et al., 2017; Jambon et al., 2019). The positive relationship between anger and reactive regression has also been supported by studies conducted among toddlers (Vitaro et al., 2006) and among Chinese adolescents (Fung et al., 2015). In addition to anger and hostility, other negative emotions have also been found to be related to reactive aggression. For example, a sizeable body of empirical studies have shown that internalizing problems, including anxiety-depression symptoms (Fung et al., 2015; Slaughter et al., 2020), chronic depression (Brendgen and Poulin, 2018; Evans and Fite, 2019), and emotional dysregulation (Stellwagen and Kerig, 2018) are significantly linked to reactive aggression.

Unlike reactive aggression that is regarded as an emotional form of aggression, proactive aggression is driven by desired goals and is characterized by lack of emotionality (Crick and Dodge, 1996). Indeed, empirical research examining the relationship between negative emotions and proactive aggression has failed to provide evidence that negative emotions act as a predictor of proactive aggression (Card and Little, 2006; Rosen and Factor, 2015). In an analysis of longitudinal data collected from a sample of 599 elementary-school children in Germany, Rohlf et al. (2017) found that anger dysregulation was positively linked to reactive aggression both concurrently and longitudinally but was not related to proactive aggression. Similarly, a more recent study conducted by Moore et al. (2019) also showed that adolescents' daily emotions including sadness, fear, and angry reactions to daily hassles are significantly linked to reactive aggression but not to proactive aggression.

Based on theories and empirical evidence reviewed in this section, we expect that negative emotions mediate the relationship between perceived discrimination and reactive aggression, but play no role linking perceived discrimination and proactive aggression. Specifically, migrant adolescents' perceived discrimination increases their experience of negative emotions, which in turn place them at a higher risk for reactive aggression.

Prior studies have shown that socioemotional support strongly influences adolescent adjustment and developmental outcomes (Ni et al., 2016; Sterle et al., 2018; Wright and Wachs, 2019). Defined as the range of behaviors through which one person conveys love (i.e., affection or emotional acceptance) and esteem (i.e., respect or social acceptance) toward another person, socioemotional support is one of the major domains of social support satisfying the most basic emotional needs of individuals (Foa and Foa, 1974). For children and adolescents who are confronted with strains, socioemotional support could facilitate a positive, resilient sense of self, and meanwhile afford an arena of comfort that would attenuate the deleterious impact of stressors (Luthar, 2006). The protective function of socioemotional support can be explained by the extension of the GST. The GST identifies social support as a protective factor, potentially alleviating the deleterious impact of strains such as discrimination by influencing an individual's subjective appraisal of strains and his or her capability to modulate emotional responses to strains (Agnew, 2013). Likewise, the theory of differential social support and coercion suggests that social support could shelter individuals from negative emotions, afford individuals resources to tackle hardship through prosocial means, thereby decreasing the adverse impact of strains like discrimination (Colvin et al., 2002). In this regard, socioemotional support as a major form of social support could play an important role in protecting migrant children from developing negative emotions and aggressive behavior.

Prior studies have provided strong evidence showing that socioemotional support can serve important protective functions, buffering individuals from adverse effects of strains. Kort-Butler (2010) found that while exposure to victimization is significantly and positively related to delinquent behavior for adolescents with lower levels of socioemotional support, the relationship does not exist for those with higher levels of socioemotional support. Utilizing data collected from African Americans, Steers et al. (2019) found that social support, in the form of socioemotional support and tangible support, moderates the relationship between discrimination and mental health problems. Similarly, in their longitudinal study of male adolescents and their parents, Simons et al. (2006) found that warmth and affection from parents mitigate the deleterious impact of discrimination on adolescent violent and aggressive behavior. Their results suggested that parental socioemotional support achieves this buffering effect by reducing the likelihood that discrimination leads to anger and hostility (Simons et al., 2006).

Based on the evidence provided in previous studies, we expect that socioemotional support moderates the relationship between discrimination and reactive aggression. Specifically, migrant children with higher level of socioemotional support are less likely to act aggressively when being discriminated against than children with lower level of socioemotional support.

Drawing on the theories and research reviewed above, this study constructs a moderated mediation model to test the mechanisms underlying the relationship between perceived discrimination and the two forms of aggression, including proactive and reactive aggression among migrant adolescents. Specifically, the current research investigates the mediating role of negative emotions and the moderating role of socioemotional support in the relationship between perceived discrimination and aggression. We use two-wave longitudinal data collected from a random sample of migrant adolescents in China to test the interrelationships in the model. We proposed the following hypotheses:

- H1: Perceived discrimination by Chinese migrant adolescents is positively related to their aggression, while its influence on reactive aggression is expected to be stronger than its effect on proactive aggression among the adolescent group.
- H2: Perceive discrimination increases negative emotions, which in turn lead to adolescent aggression, especially reactive aggression.
- H3: Socioemotional support weakens the link between perceived discrimination and aggression through the following two mechanisms: (a) it acts as a buffer to inhibit aggressive response by moderating the impact of perceived discrimination on negative emotions and (b) it suppresses aggressive response by moderating the direct effect of perceived discrimination on aggression.

## Materials and Methods

### Data

The current study used data collected from a two-wave longitudinal research project on family processes and delinquency conducted in one of the largest metropolitan areas in China. The institutional review board of the university that funded the project reviewed and approved the study design and procedures. We collected the first wave of data in 2015 and the second wave of data one year later. The research site had been a major city in China before the country opened up its economy to the world in the late 1970s, but it has developed into a highly populated and diverse metropolis in recent years with mixed urban and suburban districts. It is now home to 30 million people, including millions of migrant workers and ethnic minorities.

To ensure the representativeness of the sample, we randomly selected eligible participants who attended secondary schools designated for migrant children using a three-stage stratified probability proportionate-to-size sampling procedure. In the first stage, we randomly selected three districts to study, including two urban districts, and one suburban district. In the second stage, we randomly selected one suburban middle school, one urban middle school, one suburban high school, and one urban high school within each district, resulting in a total of 12 schools. In the third stage, in each sampled school, we proportionately selected a random number of classes in the seventh, eighth, tenth, and eleventh grades. Considering that ninth and twelfth graders, which were the final years of middle and high school, respectively, would graduate before the second wave of the survey, we did not include them in the baseline survey.

Prior to survey administration, we provided the schools with the written informed consent forms for both the students and their parents. The forms clearly state that the participation in this study is entirely voluntary, and the privacy and confidentiality of the respondents will be strictly protected. We used self-identification as a way to identify migrant students. We asked a series of questions about place of origin and adaptation to life in the city. Students were instructed to skip this set of questions if they considered themselves as a “local resident” of the city. Only respondents who answered the questions on migration were included in this study, which yielded 534 eligible participants. A paper-and-pencil survey was then administered to the sampled students. In the following year (2016), we conducted the second wave of the survey to the same students in the same schools. The response rates for the Wave 1 and Wave 2 surveys were 97.20 and 96.73%, respectively. Additionally, 61 participants who had missing values on the study variables, including the non-respondents, were excluded in the analyses, resulting in a final sample of 470.

### Measurement

We used standard instruments with verified validity and reliability in prior research to measure the key theoretical concepts introduced in this study, including perceived discrimination, negative emotions, socioemotional support, and aggression. To facilitate causal inference, aggression is measured using data collected in Time 2 (T2), while all other variables are measured by data collected in Time 1 (T1). We also included demographic measures as control variables.

#### Perceived Discrimination

Perceived discrimination was measured by Perceived Discrimination among Migrant Children (PDAMC) developed by Liu and Shen (2009). PDAMC consists of 20 five-point-Likert-scale questions measuring 4 aspects of discrimination: physical discrimination, avoidance, policy discrimination, and general discrimination. PDAMC has been widely used to assess perceived discrimination and has been shown to have good validity and reliability (Liu and Shen, 2009). The Cronbach alpha value of the items was 0.95, indicating a high level of reliability. In the current study, we measured the concept of discrimination by taking an average of all 20 questions.

#### Negative Emotions

Wang et al. (1997)'s mental health inventory of middle school and high school students (MMHI) was used to measure negative emotions in this study. We selected 30 items from five subscales (6 items each) to measure five common psychological disorder symptoms including depression (alpha = 0.84), anxiety (alpha = 0.88), interpersonal strain (alpha = 0.78), hostility (alpha = 0.86), and paranoid ideation (alpha = 0.84). For each symptom, the mean of the six items was used as a measure of the corresponding psychological disorder. An additional analysis showed that the 30 items formed a single index with an alpha value of 0.95. Considering the high level of internal consistency among the items, we took an average of the 30 items and used it as an indicator of negative emotions.

#### Socioemotional Support

Socioemotional support was measured by the Index of Sojourner Social Support (ISSS) developed by Ong and Ward (2005), which consists of 18 Likert-scaled questions. The ISSS contains separate measures of socioemotional support and instrumental support, each of which is composed of nine questions. In the current study, we used the mean score of the nine questions (alpha = 0.93) measuring socioemotional support as an indicator of the level of socioemotional support.

#### Aggression

Aggression was measured by Reactive–Proactive Aggression Questionnaire (RPQ) developed by Raine et al. (2006). The RPQ consists of 23 items, 11 for reactive aggression (alpha = 0.88) and 12 for proactive aggression (alpha = 0.94). Answer scales of the RPQ are 0 (never), 1 (sometimes), or 2 (often). Summations of the 11 and 12 items formed the measurement of reactive and proactive aggression, ranging from 0 to 22 and from 0 to 24, respectively.

Control variables. Age, gender, parental education, and family monthly income were included as the control variables in the analysis. Age was an interval variable measured by years, ranging from 11 to 17. Gender was a dichotomous variable, with 0 representing male and 1 representing female. Parental education was measured by asking the respondents to rate paternal education and maternal education separately on a 4-point scale ranging from 1 (primary school or less) to 4 (undergraduate education or more). Family monthly income was reported by the respondents on 6 categories ranging from 1 (less than RMB 1,000) to 6 (more than RMB 9,000).

### Analytical Approach

Descriptive analysis was conducted to provide an overview of the sample. After descriptive analysis, we conducted independent sample *t*-tests and *F*-tests to compare the mean differences of the key study variables by the demographic variables. Then correlational analysis was conducted to examine the bivariate relationships between the study variables. Conditional process analysis (CPA) (Hayes, 2013) was used to test the mediating effect of negative emotions and the moderating effect of socioemotional support on the relationship between perceived discrimination and the two types of aggression. We first tested the mediation model and added the interaction term to test the moderating effect of negative emotions. We applied the bootstrapping approach with 95% confidence intervals (CI) based on 2,000 random samples to estimate the effect. The 95% CI without zero indicates statistical significance (Preacher and Hayes, 2008). The CPA can be estimated by the following two regressions (Hayes, 2013):

(1) $$\begin{array}{l} \text{M}=\beta_{0}+\beta_{1}\text{X}+\beta_{2}\text{W}+\beta_{3}\text{XW}+\beta_{4}{\text{C}}_{1}\;\left(1\right) \end{array}$$

(2) $$\begin{array}{l} \text{Y}=\gamma_{0}+\gamma_{1}\text{X}+\gamma_{2}\text{M}+\gamma_{3}\text{W}+\gamma_{4}\text{XW}+\gamma_{5}{\text{C}}_{2}\;\left(2\right) \end{array}$$

In the above formulas, *X, W, M*, and *Y* denote exposure variable (independent variable), moderator, mediator, and outcome variable (dependent variable), respectively. *C*_1_ and *C*_2_ are control variables for each regression. Conventionally, the classic mediation analysis (Baron and Kenny, 1986) can be estimated by regressing X on M and regressing X + M on Y. In other words, only the first two and the last term in regression (1) and the first three and the last term in regression (2) are included in the analysis. However, in order to explore how W moderates the mediation process (the conditional process), the main effect of W and the interaction term of W and X are added to both regressions.

It should be noted that in the CPA, the parameter tests of β_3_ and γ_4_ can indicate whether W significantly moderate the effect of X on M, and the direct effect of X on Y. In classic mediation analysis, the direct, indirect and total effects of X on Y are calculated by three constants γ_1_, β_1_γ_2_, and γ_1_ + β_1_γ_2_, respectively. In contrast, because of the introduction of interactive terms, the direct, indirect and total effects of X on Y in CFA change to γ_1_ + γ_4_*W*, (β_2_ + β_3_*W*)γ_2_, and γ_1_ + γ_4_*W* + (β_2_ + β_3_*W*)γ_2_, which are not constant but determined by the values of W. The interpretation of the direct, indirect, and total effects, therefore, is usually done by substituting the average value and the average value minus/plus 1 standard deviation of W as representatives of medium, low or high levels of the moderator into the equations to predict the contextual direct, indirect, and total effects.

## Results

Table 1 provides the descriptive statistics of all the variables included in the analysis. As shown in the table, 46.17% of the surveyed migrant students were female and the average age was 13.49. More than half of the respondents' family monthly income were between 3,001 and 5,000 RMB (52.34%). Most of respondents' parents received secondary school education. The sample as a whole reported experiencing a modest level of perceived discrimination and a medium level of socioemotional support. The average score of negative emotions exceeded 2. Wang et al. (1997) has suggested that a mean score of 2 indicates the presence of a mild level of mental illness. Based on their suggestion, we calculated the prevalence rate of negative emotions among migrant adolescents in our sample, which was 46.81%, suggesting that 46.81% of migrant adolescents suffered from some level of mental health problems. Furthermore, the mean score of proactive aggression and reactive aggression was 2.23 and 5.44, respectively, indicating that reactive aggression was nearly one and a half times higher than proactive aggression among Chinese migrant students.

**Table 1:** Descriptive statistics (*N* = 470).

**Variable**	**Mean/%**	**Std. Dev.**	**Min**	**Max**
Perceived discrimination	1.60	0.71	1	5
Socioemotional support	2.89	1.00	1	5
Negative emotions	2.09	0.78	1	5
*Aggression*				
Proactive aggression	2.23	3.94	0	24
Reactive aggression	5.44	4.25	0	22
*Demographics*				
Gender				
Male	53.83%			
Female	46.17%			
Age	13.49	1.47	11	17
Family monthly income				
<1,000	1.70%			
1,000–3,000	21.19%			
3,001–5,000	52.34%			
5,001–7,000	13.40%			
7,001–9,000	5.96%			
>9,000	4.68%			
Paternal education				
Primary school or less	18.51%			
Secondary school	75.11%			
Junior college	4.47%			
Undergraduate education or more	1.91%			
Maternal education				
Primary school or less	25.53%			
Secondary school	69.57%			
Junior college	3.62%			
Undergraduate education or more	1.28%			

Table 2 presents the mean differences of the key study variables by gender, family monthly income, paternal education and maternal education. There are significant gender differences in perceived discrimination, socioemotional support, proactive aggression, and reactive aggression. Compared with their female counterparts, male adolescents reported higher levels of perceived discrimination (male: *M* = 1.70; female: *M* = 1.48; *t* = 3.28, *p* < 0.001), proactive aggression (male: *M* = 3.13; female: *M* = 1.18; *t* = 5.53, *p* < 0.001), reactive aggression (male: *M* = 5.92; female: *M* = 4.91; *t* = 2.58, *p* < 0.05), and had a lower level of socioemotional support (male: *M* = 2.77; female: *M* = 3.03; *t* = −2.75, *p* < 0.01). There was no significant difference in study variables by family monthly income, paternal education, and maternal education.

**Table 2:** Descriptive statistics by demographic variables (*N* = 470).

**Variable**	**Perceived discrimination**	**Socioemotional support**	**Negative emotions**	**Proactive aggression**	**Reactive aggression**
**Gender**					
Male	1.70	2.77	2.07	3.13	5.92
Female	1.48	3.03	2.12	1.18	4.91
*T* statistics	3.28^***^	−2.75^**^	−0.71	5.53^***^	2.58^*^
**Family monthly income**					
<1,000	2.00	2.68	2.69	1.13	5.25
1,000–3,000	1.61	2.81	2.05	1.97	5.07
3,001–5,000	1.60	2.93	2.09	2.24	5.38
5,001–7,000	1.61	2.75	2.00	2.30	6.02
7,001–9,000	1.58	3.05	2.35	2.61	6.00
>9,000	1.34	3.12	2.00	3.00	5.82
*F* statistics	1.11	0.87	1.88	0.44	0.53
**Paternal education**					
Primary school or less	1.63	2.86	2.20	2.45	5.78
Secondary school	1.61	2.89	2.06	2.22	5.39
Junior college	1.41	2.79	2.23	1.90	5.58
Undergraduate or more	1.21	3.30	2.07	1.33	4.56
*F* statistics	1.46	0.58	1.01	0.29	0.34
**Maternal education**					
Primary school or less	1.61	2.90	2.10	2.58	5.87
Secondary school	1.61	2.85	2.09	2.19	5.36
Junior college	1.19	3.21	2.04	1.06	5.18
Undergraduate or more	1.76	3.63	2.37	0.83	2.67
*F* statistic	2.02	1.82	0.28	1.07	1.31

* *P < 0.05*;

** *p < 0.01*;

*** *P < 0.001*.

Table 3 shows the Pearson correlation coefficients for all the bivariate relationships among the key variables included in the analysis, which provide a partial test of our research hypotheses. As shown in the table, perceived discrimination was positively correlated with proactive aggression (*r* = 0.12, *p* < 0.05) and negative emotions (*r* = 0.26, *p* < 0.001). Table 3 also shows that perceived discrimination was negatively correlated with socioemotional support (*r* = −0.23, *p* < 0.001). Further, negative emotions were also positively correlated with proactive (*r* = 0.10, *p* < 0.05) and reactive (*r* = 0.18, *p* < 0.001) aggression. Finally, socioemotional support was negatively associated with proactive (*r* = −0.12, *p* < 0.05) and reactive (*r* = −0.10, *p* < 0.05) aggression.

**Table 3:** Zero-order correlations.

		**1**	**2**	**3**	**4**	**5**
1	Perceived discrimination	1				
2	Socioemotional support	−0.23^***^	1			
3	Negative emotions	0.26^***^	−0.13^**^	1		
4	Proactive aggression	0.12^*^	−0.12^*^	0.10^*^	1	
5	Reactive aggression	0.09	−0.10^*^	0.18^***^	0.69^***^	1

*All correlation coefficients are standardized*.

* *P < 0.05*;

** *p < 0.01*;

*** *P < 0.001*.

**Figure 1:**
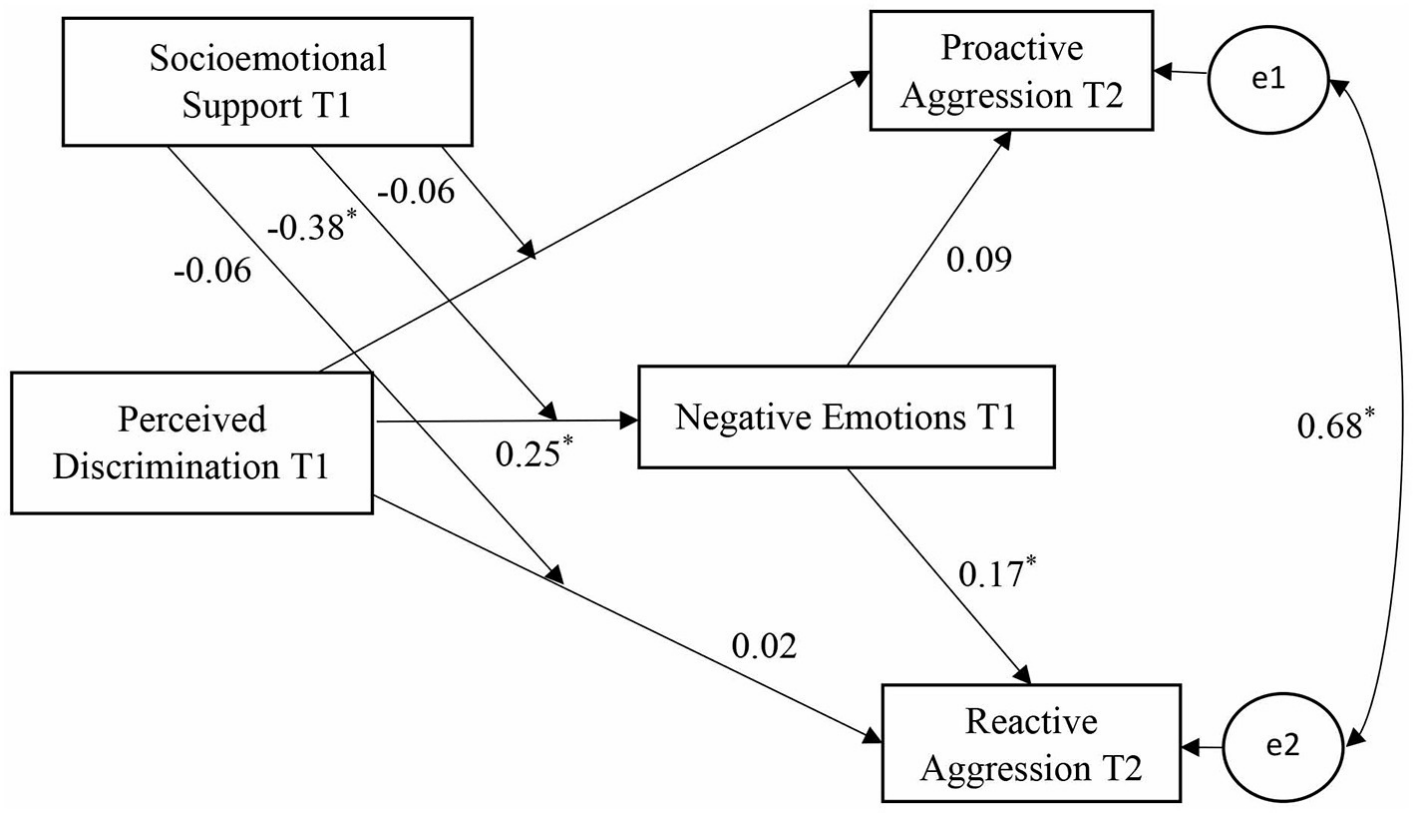
The conditional process analysis. All the coefficients are standardized. Gender, age, family monthly income and parental education are controlled for all the endogenous variables. *P < 0.05.

As Pearson correlations only reflect bivariate relationships between the variables, a CPA was conducted to provide more robust evidence for the research hypotheses. Figure 1 shows the framework and standardized coefficients of the CPA analysis.

As indicated in model 1 in Table 4, the direct effects of perceived discrimination on proactive/reactive aggression were not significant. However, the effect of perceived discrimination on negative emotions was significant (β = 0.25, bootstrapping 95% CI = [0.13, 0.38]), and the effect of negative emotions on reactive aggression was also significant (β = 0.17, bootstrapping 95% CI = [0.06, 0.28]). These findings supported the first two hypotheses that perceived discrimination was significantly related to reactive aggression and its effect was mediated by negative emotions. The effect of perceived discrimination on proactive aggression, however, was not significant.

**Table 4:** The results of conditional process analysis.

**Variables**	**Model 1**	**Model 2**
**Endogenous variables: Negative emotions**		
Gender	0.08(−0.01 to 0.18)	0.09(−0.01 to 0.18)
Age	0.05(−0.04 to 0.13)	0.05(−0.03 to 0.14)
Family monthly income	0.03(−0.07 to 0.13)	0.03(−0.06 to 0.13)
Paternal education	−0.02(−0.12 to 0.07)	−0.03(−0.13 to 0.06)
Maternal education	0.02(−0.06 to 0.11)	0.03(−0.06 to 0.11)
Perceived discrimination	0.25(0.13 to 0.38)^*^	0.57(0.28 to 0.87)^*^
Socioemotional support	−0.08(−0.20 to 0.04)	0.19(−0.09 to 0.46)
Discrimination × support		−0.38(−0.72 to −0.05)^*^
Intercept	1.71(0.69 to 2.75)	0.96(−0.28 to 2.20)
**Endogenous variables: Proactive aggression**		
Gender	−0.23(−0.30 to −0.16)^*^	−0.23(−0.30 to −0.16)^*^
Age	−0.02(−0.09 to 0.05)	−0.02(−0.09 to 0.06)
Family monthly income	0.06(−0.03 to 0.16)	0.06(−0.03 to 0.16)
Paternal education	−0.02(−0.11 to 0.07)	−0.02(−0.11 to 0.06)
Maternal education	−0.07(−0.15 to 0.00)	−0.07(−0.15 to 0.00)
Negative emotions	0.09(−0.02 to 0.20)	0.09(−0.03 to 0.20)
Perceived discrimination	0.04(−0.07 to 0.15)	0.09(−0.26 to 0.44)
Socioemotional support	−0.07(−0.18 to 0.04)	−0.03(−0.28 to 0.22)
Discrimination × support		−0.06(−0.41 to 0.29)
Intercept	0.95(−0.02 to 1.93)	0.84(−0.43 to 2.11)
**Endogenous variables: Reactive aggression**		
Gender	−0.10(−0.19 to −0.02)^*^	−0.10(−0.19 to −0.01)^*^
Age	0.01(−0.07 to 0.09)	0.01(−0.07 to 0.09)
Family monthly income	0.08(−0.01 to 0.17)	0.08(−0.01 to 0.17)
Paternal education	−0.01(−0.10 to 0.09)	−0.01(−0.10 to 0.09)
Maternal education	−0.09(−0.18 to −0.00)^*^	−0.09(−0.18 to −0.00)^*^
Negative emotions	0.17(0.06 to 0.28)^*^	0.17(0.06 to 0.28)^*^
Perceived discrimination	0.02(−0.09 to 0.12)	0.07(−0.22 to 0.35)
Socioemotional support	−0.06(−0.16 to 0.04)	−0.02(−0.23 to 0.20)
Discrimination × support		−0.06(−0.36 to 0.23)
Intercept	1.07(0.07 to 2.06)^*^	0.95(−0.22 to 2.12)
Covariance (proactive vs. reactive)	0.68(0.61 to 0.74)	0.68(0.62 to 0.74)

*All the coefficients are standardized. Bootstrapping 95% CI with 2,000 replicates are shown in parenthesis*.

* *Indicates that bootstrapping CI does not include zero*.

Model 2 in Table 4 added the interaction term (perceived discrimination × socioemotional support). Model 2 revealed that socioemotional support significantly moderated the relationship between perceived discrimination and negative emotions (β = −0.38, bootstrapping 95% CI = [−0.72,−0.05]). Through this mechanism, socioemotional support moderated the total effect of perceived discrimination on reactive aggression by reducing the effect of discrimination on negative emotions. The direct effects of perceived discrimination on both proactive and reactive aggression, however, were not moderated by socioemotional support. These findings support hypotheses H3a and H3b.

**Figure 2:**
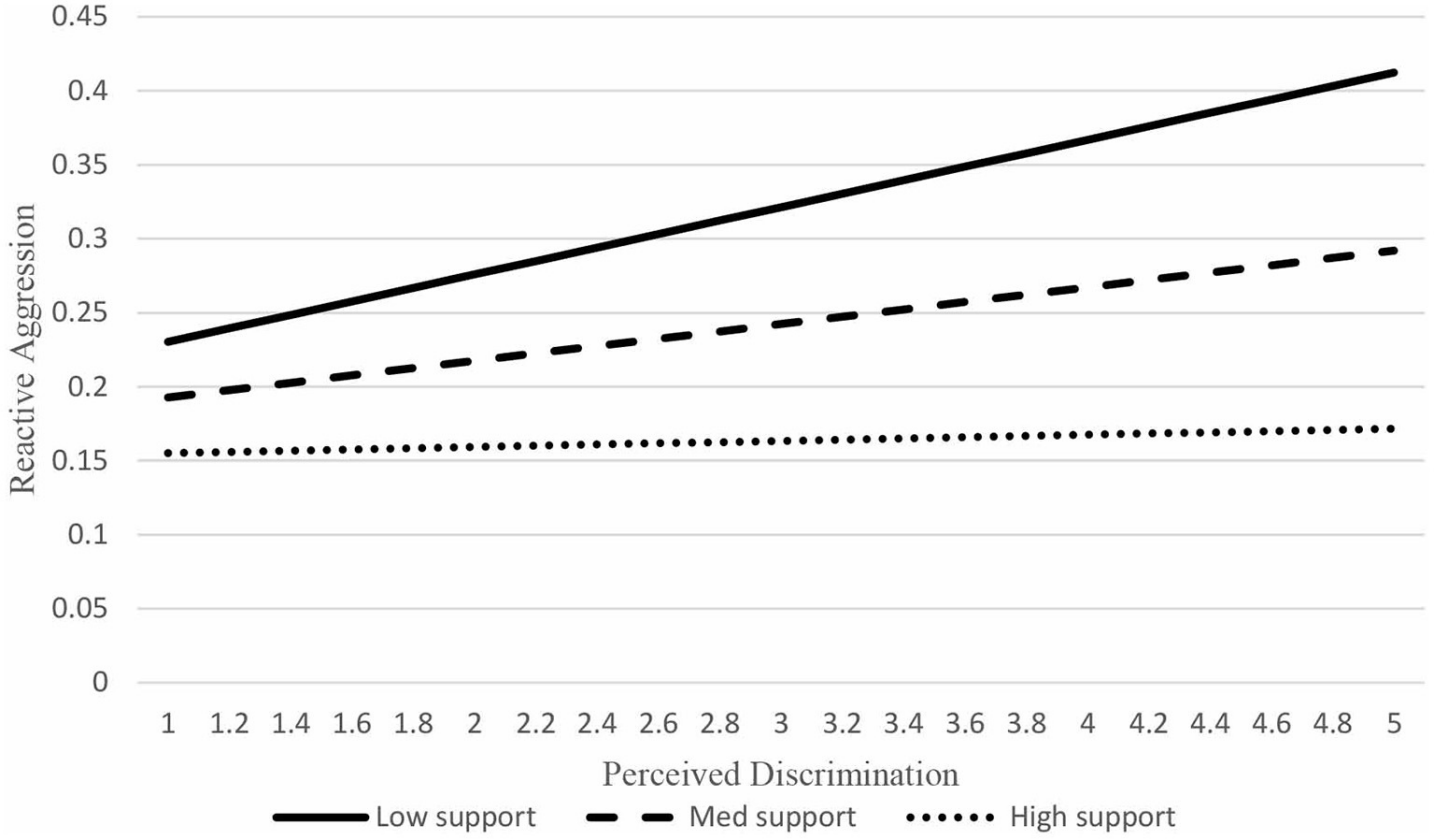
The moderating effect of socioemotional support.

To facilitate the interpretation of the moderating effect of emotional support, Figure 2 plots the total effect of perceived discrimination on reactive aggression at different levels of socioemotional support with simulated data. The low, medium, high levels of emotional support were defined by the average minus one standard deviation, the average, and the average plus one standard deviation of the measure of socioemotional support. We firstly simulated a set of values from 1 to 5 by 0.2 to represent perceived discrimination, and then calculated the corresponding value of reactive aggression based on the model described in Table 4 and Figure 1. As shown in Figure 2, although the total effects of perceived discrimination on reactive aggression were positive at all levels of socioemotional support, the slop descended when socioemotional support changed from high to low, suggesting that adolescents perceiving discrimination were less likely to engage in reactive aggression when they had higher levels of socioemotional support.

## Discussion

The relationship between perceived discrimination and antisocial behavior has garnered considering research interest in recent year. Much of this research has focused on the link between discrimination and illegal behavior. Few studies have examined the influence of discrimination on the broad range of aggressive behavior common among adolescents, including proactive and reactive aggression. The current research fills this gap. Using longitudinal data collected from a representative sample of migrant adolescents in one of the largest urban areas in China, this study found that perceived discrimination was differentially related to reactive aggression and proactive aggression. Specifically, the results showed that perceived discrimination in Wave 1 increased reactive aggression in wave 2 but it had no effect on proactive aggression in the second wave. These findings are consistent with the propositions of the GST (Agnew, 1992, 2013) and the frustration-aggression model (Berkowitz, 1962), suggesting that exposure to discrimination promote various circumstances in which children would act out and cope with provocations aggressively. As a reaction to provocations, reactive aggression may be regarded as an effective mean by those who have experienced discrimination to defend themselves, take revenge on the perpetrator and unleash anger triggered by discrimination (Berkowitz, 1962; Simons et al., 2003). The positive relationship between discrimination and reactive aggression is also in line with Dodge (1980) finding that children who are mistreated by a peer tend to attribute a hostile intention to the instigator of the unfair treatment, which in turn foster a belief that reactive aggression is a necessary and justifiable response. Consistent with our hypothesis, perceived discrimination was not significantly related to proactive aggression. This finding confirms the previous evidence that stressful life events did not predict proactive aggression (Fite et al., 2012; Brown et al., 2016), suggesting that discrimination might affect reactive aggression only.

The differential relations of perceived discrimination with reactive aggression and proactive aggression are believed to be attributed to the mechanisms underlying these relationships. Moving beyond the identification of the overall association between perceived discrimination and the two types of aggression, the current study takes a step further to examine the mediating role of negative emotions in the formation of the relationships. The results showed that negative emotions fully mediated the relationship between perceived discrimination and reactive aggression. Exposure to discrimination appeared to interfere with adolescents' ability to regulate their emotions, which in turn led to reactive aggression (Fite et al., 2012). This finding provides further support of the GST's view that the association between discrimination and aggression is indirect through negative emotions. However, the process was true only for reactive aggression as perceived discrimination had no effect on proactive aggression. Taken together, these findings suggested that both discrimination and negative emotions contributed to reactive aggression, with the former playing a more distal role and the latter a more proximate role in facilitating the behavior.

Furthermore, we investigated the buffering role of socioemotional support in moderating the relationship between perceived discrimination and the two types of aggression. In support of the argument that social support inhibits aggressive and delinquent behavior (Agnew, 1992, 2013; Colvin et al., 2002), our result confirmed that socioemotional support mitigated the adverse effect of discrimination on adolescent reactive aggression. Socioemotional support that fulfills the basic emotional needs of individuals is thought to promote a positive and resilient sense of self (Luthar, 2006). Therefore, even in the presence of stressors like discrimination, migrant adolescents with high levels of socioemotional support may be able to cope with stressors with socially appropriate behavior rather than involving in reactive aggression. We further examined the mechanisms through which socioemotional support achieves this buffering effect. We found that socioemotional support fulfills this role by reducing the likelihood that perceived discrimination leads to negative emotions. When facing discrimination, migrant adolescents with a higher level of socioemotional support were less likely to develop negative emotions. As suggested by the theory of differential social support and coercion and the GST, it appeared likely that socioemotional support such as warmth and care reduced the emotional impact of discrimination by enabling migrant adolescents to reevaluate the strain of discrimination and cope with it by non-threatening means, thereby leading to a decreased likelihood of aggressive response (Agnew, 2013).

The findings of the current study have important implications for social policy and programs related to aggression prevention and intervention. First, considering the critical role that socioemotional support plays in protecting migrant adolescents from negative emotions and aggressive behavior, it is important to provide migrant adolescents with strong socioemotional support when they resettle in big cities. For example, in the family context, parent service programs could be afforded to migrant workers to improve their awareness that socioemotional support is important for their children's development and well-being. It would also be helpful to make more time available to migrant workers for them to attend to their children's social and emotional needs. Second, schools should make a strong effort to promote multiculturism and foster a climate of mutual support and inclusiveness. For example, teachers can help migrant children develop friendship with local students and promote positive interactions between them to increase their mutual respect and trust. Research has demonstrated that building peer support system is the most effective way to improve migrant adolescents' post-settlement adjustment (Wu et al., 2010). Third, prevention programs should be provided to at-risk adolescents, especially those who have been frequently targeted for discrimination by their peers, to help them learn constructive and problem-solving coping strategies to deal with interpersonal conflict, thereby preventing them from repeated discrimination victimization and adaptation of aggressive responses that may further undermine their well-being. Lastly, active measures should be taken at the policy level to curtail discrimination. The lack of social resources and equal education opportunities caused by policy discrimination against migrant families and children is the hardest to resolve, because it stems from the rigidity of the household registration system that is deeply rooted in Chinese social structure (Wang and Mesman, 2015). Despite the structural barriers, government on all levels can make policy or regulatory changes to allot more resources for migrant adolescents, such as constructing a favorable and supportive community environment, expanding educational opportunities, and bridging the gap between regular public schools and schools designated for migrant children. The joint efforts of eliminating discrimination and building support system from family, school, and government will decrease migrant adolescents' risk for reactive aggression and promote their post-settlement well-being.

Despite the contributions of the current study, there are certain limitations that are worth noting. Firstly, aggression at Time 1 and Time 2 might be highly correlated. The survey, however, did not collect data on aggression in Wave 1. Because of it, this study was unable to control for aggression at Time 1. The omission of the Time 1 measure might have affected the accuracy of the research findings concerning the relationships between the explanatory variables and aggression. Second, although the sample adopted in this study was randomly selected, it may not be representative of all migrant children in China because cities may differ in their policies and regulations for migrant children. As such, the patterns observed in this study may not be applicable to other cities in China. To address these limitations, future studies should consider incorporating measures from multi-informants such as teachers, parents, and peers to reduce measurement errors. Additionally, to yield more unbiased and reliable results, researchers should include measures of aggression across waves in their analysis. Moreover, further research should be conducted in different cities to strengthen the external validity of the research findings.

## Data Availability Statement

The dataset used in this paper is not publicly available because of data use restrictions from the university that sponsored the study. Requests to access the dataset should be directed to SL at spencerli@um.edu.mo.

## Ethics Statement

The studies involving human participants were reviewed and approved by Research Ethics Committee, University of Macau. Written informed consent to participate in this study was provided by the participants' legal guardian/next of kin.

## Author Contributions

SL conceived the project, obtained the funding, contributed to data collection and the design of the study, reviewed and edited the manuscript. RX contributed to the design of the study, data interpretation, drafted the original manuscript, and revised it based on the critical comments provided by SL. YX conducted formal analysis and drafted the original manuscript. All authors have read and agreed to the published version of the manuscript.

## Conflict of Interest

The authors declare that the research was conducted in the absence of any commercial or financial relationships that could be construed as a potential conflict of interest.
